# Characterizing Self-Reported Tobacco, Vaping, and Marijuana-Related Tweets Geolocated for California College Campuses

**DOI:** 10.3389/fpubh.2021.628812

**Published:** 2021-04-13

**Authors:** Raphael E. Cuomo, Vidya L. Purushothaman, Jiawei Li, Cortni Bardier, Matthew Nali, Neal Shah, Nick Obradovich, Joshua Yang, Tim K. Mackey

**Affiliations:** ^1^Department of Anesthesiology, San Diego School of Medicine, University of California, San Diego, San Diego, CA, United States; ^2^Global Health Policy and Data Institute, San Diego, CA, United States; ^3^S-3 Research, San Diego, CA, United States; ^4^Center for Humans and Machines, Max Planck Institute for Human Development, Berlin, Germany; ^5^Department of Public Health, California State University, Fullerton, Fullerton, CA, United States

**Keywords:** infoveillance, tobacco, Twitter, surveillance, college, marijuana, vaping, policy

## Abstract

**Introduction:** College-aged youth are active on social media yet smoking-related social media engagement in these populations has not been thoroughly investigated. We sought to conduct an exploratory infoveillance study focused on geolocated data to characterize smoking-related tweets originating from California 4-year colleges on Twitter.

**Methods:** Tweets from 2015 to 2019 with geospatial coordinates in CA college campuses containing smoking-related keywords were collected from the Twitter API stream and manually annotated for discussions about smoking product type, sentiment, and behavior.

**Results:** Out of all tweets detected with smoking-related behavior, 46.7% related to tobacco use, 50.0% to marijuana, and 7.3% to vaping. Of these tweets, 46.1% reported first-person use or second-hand observation of smoking behavior. Out of 962 tweets with user sentiment, the majority (67.6%) were positive, ranging from 55.0% for California State University, Long Beach to 95.8% for California State University, Los Angeles.

**Discussion:** We detected reporting of first- and second-hand smoking behavior on CA college campuses representing possible violation of campus smoking bans. The majority of tweets expressed positive sentiment about smoking behaviors, though there was appreciable variability between college campuses. This suggests that anti-smoking outreach should be tailored to the unique student populations of these college communities.

**Conclusion:** Among tweets about smoking from California colleges, high levels of positive sentiment suggest that the campus climate may be less receptive to anti-smoking messages or adherence to campus smoking bans. Further research should investigate the degree to which this varies by campuses over time and following implementation of bans including validating using other sources of data.

## Introduction

College-going individuals in the United States may have unique attitudes toward substance use behavior and tobacco use, including shifts in attitudes and behaviors that are associated with the constantly changing product landscape of alternative tobacco products (ATPs), such as electronic-cigarettes (1, 2). Psychosocial behaviors and campus culture, including class attendance, peer socializing, campus policies, and residential environments, may also facilitate these unique attitudes toward favorability of smoking among college subgroups, while also introducing a unique risk environments for tobacco initiation, uptake, transition, and use (3–5). In addition, part of the variation explaining these health behaviors may be influenced by the specific demographic and socioeconomic characteristics of a college campus population and community.

Data from social media platforms are often used to self-report and publicly communicate health-related attitudes and behaviors (6). Young adults [ages 18–25 (7)] in the United States are much more likely than older populations to actively use social media, including popular platforms Twitter, Snapchat, and Instagram (8). Infoveillance research, which uses online information sources to detect trends about the distribution and determinants of disease, including health knowledge and behaviors, has been used to develop insights on numerous public health issues including infectious diseases, vaccination sentiment, opioid use disorder, mental health issues, and, relevant to the exploratory aims of this study, tobacco and alternative tobacco use attitudes and behavior (9–11).

However, smoking-related discussions on social media tied to specific colleges with geographic specificity has not been widely investigated. Existing studies using social media to examine tobacco-related attitudes and behaviors in college-aged populations have primarily focused on evaluating the impact of social media health promotion anti-tobacco campaigns, recruiting hard-to-reach college populations using social media platforms, and examining the influence of exposure to tobacco-related social media content and marketing on current and future behavior and use (12–16). Other research (e.g., surveys, focus groups, etc.) on college-aged populations has focused on assessing tobacco initiation and transition of use patterns, particularly as new alternative and emerging tobacco products become available (17, 18). Accelerating research using social media to assess tobacco-related attitudes/influences among youth has also been supported by U.S. Federal initiatives, including projects funded by the National Cancer Institute and U.S. Food and Drug Administration Tobacco Centers of Regulatory Science, which for have identified and characterized e-cigarette advertisements on image-focused social media sites and tobacco user experiences with little cigars and e-cigarettes as discussed on Twitter (19–23).

Changes in local, state, and national health policy related to tobacco and other products smoked or used concurrently with tobacco and electronic cigarettes can also have an impact on attitudes and behaviors of these populations. For example, recent debate in the United States regarding the legalization of marijuana/cannabis may positively influence marijuana-related attitudes for college populations, who tend to skew toward more liberal policies regarding decriminalization, legalization, and increased access (24). Similarly, the 2019 outbreak of e-cigarette and vaping-related lung injury (EVALI) associated with products containing tetrahydrocannabinol (THC) may dissuade tobacco or THC use in certain young adult populations, particularly since they were most heavily impacted by the disease (25).

Examining the changing public attitudes and behaviors of college-aged smokers is particularly salient for the State of California, USA. As of January 2014, all campuses in the statewide University of California (UC) system became tobacco-free (26), and the California State University (CSU) system followed suit in 2017 (27). In addition, voters in California approved Proposition 56 in late 2016, which added a $2.00 increase to the cigarette tax effective April 2017, with an equivalent increase on other tobacco products and electronic cigarettes (28). Voters in 2016 also approved Proposition 64, which legalized the use of recreational cannabis in November 2016 (29). During this time, the popularity of e-cigarettes in the United States was increasing (30). These changes in policy and preferences underscore the interconnected nature of the Triangulum of tobacco products (tobacco, marijuana, and e-cigarettes), including potential for dual-use, transition between products, and challenges associated with conducting surveillance and implementing cessation programs (31, 32).

This changing policy landscape supporting tobacco control measures, as reflected in the shift of California's public university systems to become smoke-free, is a key impetus for this study. The ability of these colleges to eliminate on-campus smoking relies in large part on understanding past and existing knowledge and attitudes held by the campus smoking populations, along with their perceptions and behaviors that may be associated with compliance or non-compliance to smoke free campus policies. In response, this study conducted exploratory research on the popular microblogging platform Twitter. Specifically, we used big data, data mining, and geospatial approaches to identify and characterize tweets originating from Twitter users specifically geolocated at California 4-year university campuses. Our primary objective was to assess types of tobacco and ATP products mentioned by users, the distribution of user sentiment toward tobacco and smoking behavior, and to assess the feasibility of detecting self-reported smoking behavior that may represent a violation of campus smoke free policies. Secondarily, we also sought to conduct a cross-campus assessment to determine how these factors vary across different university and college communities and over time.

## Methods

### Data Collection

The objective of the study's data collection approach was to obtain a highly refined subset of tweets, which were both posted from college campus' geolocated coordinates in California and also included user discussions about smoking, in preparation for manual review to more purposefully identify tweets that specifically discussed different types of tobacco and smoking products, sentiment of users toward smoking behavior, and self-reported smoking behavior on campus. Data were collected from the Twitter public streaming Application Programming Interface (API) using the cloud-computing service Amazon Web Services (AWS). The public streaming API was set with filters to collect all tweets that included metadata containing latitude and longitude coordinates, initially with no filter for keywords. Tweets were collected continuously from 2015 to 2019. All tweets collected included the text of the tweet and associated metadata, including the date and time of tweets.

The use of the public Twitter streaming API to collect data pre-filtered only for tweets including latitude and longitude coordinates represent a subset of all tweets posted during the time frame of the study. There exists the potential for sampling bias associated with different Twitter APIs that are not representative of all Twitter data (e.g., Firehose data), and data filtered only for geocoded data may omit many conversations from college-aged populations about topics, such as smoking, which may be linked to college-related user groups (see “Limitations” section for more details) (50). Though resulting in a much smaller volume of data, our approach nevertheless allows for detection of tweets in specific geospatial bounds at the high resolution of latitude and longitude coordinates in the state of California. Therefore, by using this data collection approach, we were able to isolate tweets originating from geospatial coordinates within the formal spatial boundaries of all 4-year universities in California. To enable this geolocation, a basemap of California 4-year universities from the Stanford Prevention Research Center (SPRC) was obtained and cross-referenced. Tweet geolocated points were spatially joined to campus polygons using ArcGIS software. The SPRC's basemap included a relational geodatabase which classified polygons by college name. College areas were comprised of multiple polygons for different campuses and associated properties, though aggregation was conducted at the overall college level to enable comparison across different colleges.

Tweets were then filtered for 37 keywords which were broadly related to tobacco-related topics, including the names and brands of different tobacco and ATPs and descriptive terms associated with smoking and vaping as expanded upon from those used in prior studies (9, 33). Specifically, the following keywords were used: bidis, cigarette, cigarettes, cigarillos, cigars, cigie, class, dip, e-cig, hookah, huqqa, joint, JUUl, kereteks, Marlboro, Newport, njoy, pipe, roll-up, shag, smoke, smoking, snuff, snus, tobacco, vape, vaped, vapejuice, vaper, vapes, vaping, vapor, waterpipe, waxpen, and weed. The purpose of this keyword filtering was to better isolate smoking-related conversations from all other Twitter discussions occurring on college campuses. After tweets were manually reviewed to positively identify smoking-related conversations originating in these college campuses, a snowball sampling design was employed which compared the frequency of all non-keyword terms in “signal” tweets (i.e., tweets that were confirmed via manual annotation to be associated with smoking products and behavior) with the frequency of these words in “noise” tweets (i.e., tweets that were unrelated to smoking). This methodology resulted in the identification and querying for ten additional keywords: 420, 818, blunt, bong, cigs, kush, marijuana, roll, smell, and stoge.

### Data Analysis

After isolating a corpus filtered for tobacco-related keywords in areas geolocated for California 4-year universities, four researchers trained in social media content analysis used an inductive coding approach to identify study characteristics of interest by manually annotating all tweets (i.e., all tweets identified by keyword search were read by four different researchers), following an approach also described in prior studies (33–37). Annotators had backgrounds in public health and had experience manually annotating social media posts for tobacco behaviors in prior published research projects (9, 33). Manual annotation included: (a) identifying the type of smoking product discussed (i.e., marijuana, tobacco, vape/e-cigarette); (b) assessing positive, negative, or neutral sentiment related to smoking behavior (e.g., users expressing positive attitudes/beliefs about vaping); and (c) identification of whether the tweet included first-person use or second-hand observation of smoking behavior.

Table A1 contains further details about topics that were coded as valid and invalid for positive identification as a “signal” tweet. Tweets that did not express sentiment related to smoking were excluded from analysis of signal tweets. The primary objective of this approach was to conduct exploratory research into what tobacco and smoking products were being discussed by Twitter users at California universities, assess the overall sentiment toward tobacco and smoking by these users, and explore whether it was possible to identify self-reporting of tobacco use-related behavior (which could constitute a violation of smoke free campus policies). Four authors (VP, CB, MN, and NS) coded posts independently and achieved a high interrater reliability for overall coding categories (kappa = 0.96) and equally high interrater reliability for specific sub-coding for tobacco (kappa = 0.96), vape (kappa = 0.96), and marijuana (kappa = 0.95) specific tweet categories. For inconsistent results and any discrepancies related to coding, all authors convened to discuss, confer, and reach consensus on the correct classification informed by the inductive coding approach outlined in Table A1.

Analyses of variation across college campuses were limited to the top twenty colleges by tweet volume, as estimates collected from samples of tweets from other colleges may have been biased due to insufficient volume of tweets collected. A *p* < 0.10 was considered statistically significant for correlational analyses due to sample size limitation. Point density algorithms were used to visualize and detect geospatial trends. Analysis was conducted in R version 4.0.1 and geospatial visualization of data was done in ArcGIS Desktop version 10.7. This project was part of a broader study to examine college campus smoke-free policies using qualitative focus groups and examining social media data with the qualitative analysis approved by the Institutional Review Board at California State University, Fullerton (HSR-18-19-532).

## Results

Data collection resulted in 83,723,435 geo-identifiable tweets located in the state of California in the 5-year period from 2015 to 2019. From these tweets, 1,381,019 (1.6%) originated from 88 CA 4-year colleges, with the five schools contributing the most tweets including UC Los Angeles (138,979), Stanford University (70,831), UC Riverside (68,103), University of Southern California (65,600), and UC Berkeley (49,911). Thirty-eight schools contributed over 10,000 tweets each, overall representing 89% of the entire corpus of CA 4-year college geocoded tweets. Of these tweets, 7,342 (0.53%) contained smoking-related keywords with approximately one-third occurring after 2015. In total, smoking-related topics originating from all geocoded tweets in the state made up an extremely small proportion of all topics and tweets specifically geocoded for CA 4-year universities. This low representation of smoking-related twitter topics likely was impacted by the methodology of data collection and its associated limitations (see “Limitations” section).

Of the 34 smoking-related keywords used to query the Twitter API, eight returned over 100 tweets from college campuses during this time period: cigarette (*n* = 123), dip (866), joint (212), njoy (2,611), pipe (212), smoke (877), smoking (255), and weed (638). Upon further examination, it was determined that “njoy” returned tweets with the word “enjoy” in 99.2% of cases, and “dip” also predominantly returned false positives. After excluding these two terms, longitudinal analysis revealed that the rate of “weed” in the corpus decreased through the study time period, with it being found in 28% of tweets in 2015, 15% in 2016, 10% in 2017, 14% in 2018, and 7% in 2019. Conversely, the rate of “pipe” in the corpus increased from 7% in 2015 to 14% in 2016, 16% in 2017, 14% in 2018, and 12% in 2019. Also notable was the rate of “joint,” which increased from 6% in 2015 to 15% in 2016, 19% in 2017, 16% in 2018, and 14% in 2019. The frequency of these keywords may have been impacted by changes in the way users communicate about smoking-related topics, in addition to the potential impact of legalization of adult-use cannabis in 2016. Other terms were comparatively stable, with “smoke” returning the top number of tweets in the corpus for any given year in the study period.

**Figure 1:**
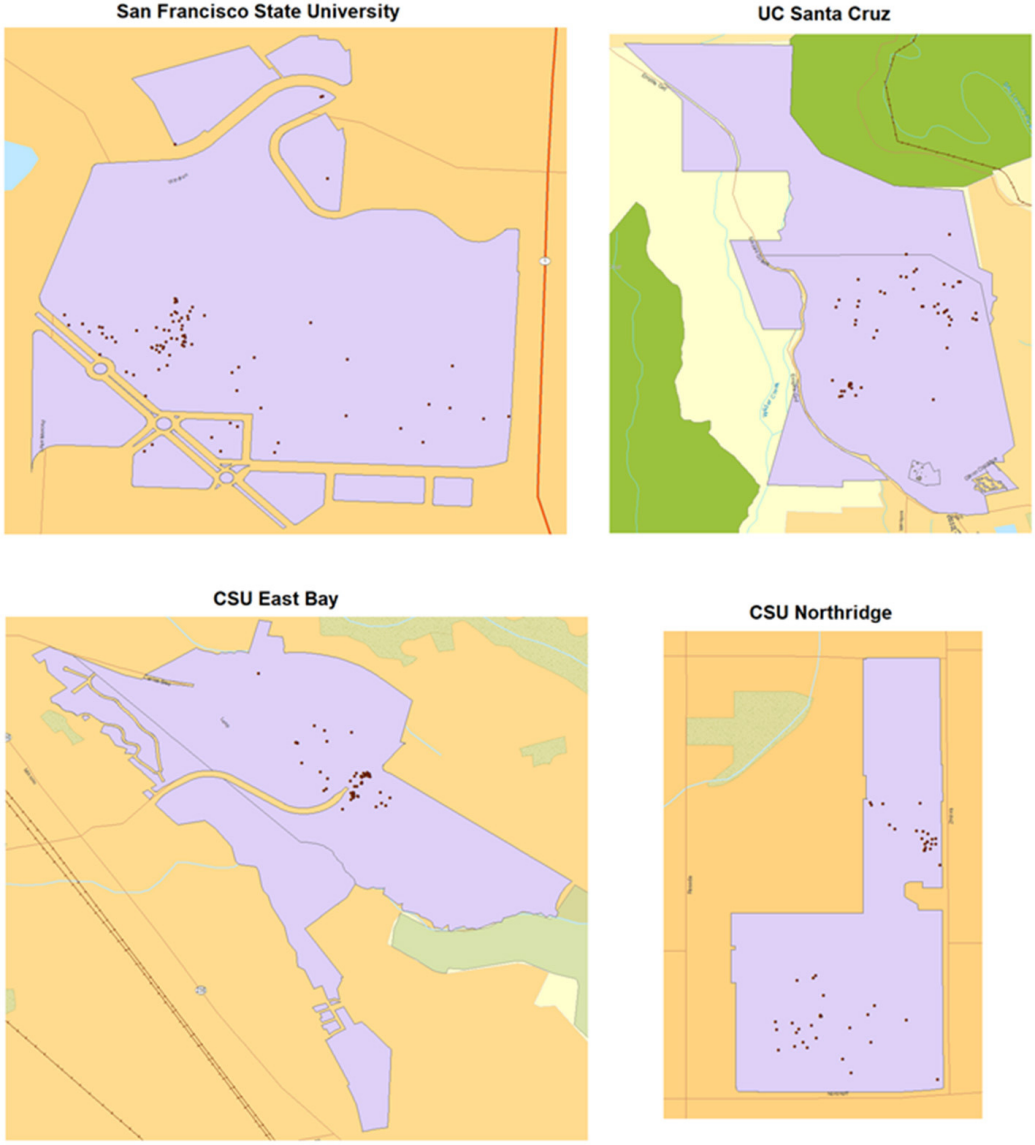
Locations of Twitter posts from the top four colleges by volume of smoking-related tweets, with purple polygons denoting university lands, and dots representing tweet locations.

From this subset of filtered geocoded data, manual review identified 1,089 “signal” tweets relating directly to smoking topics, with 509 (46.7%) relating to tobacco, 490 (50.00%) relating to marijuana, 79 (7.3%) relating to vaping, and 7 relating to multiple product types in the same tweet (0.6%). Sixty-eight CA colleges were represented in our signal data, though the top 20 accounted for 783 (71.90%) of tweets. Individual colleges exhibited high variation in the proportion of tweets corresponding to each smoking product assessed (see Table 1 and Figure 1). Out of the top twenty colleges by tweet volume, the distribution of tobacco-related tweets ranged from 26.1% for CSU Long Beach to 62.2% for CSU San Jose [median [M] = 43.1%, standard deviation [SD] = 10.7%]. Vaping-related tweets were detected from eighteen of these twenty colleges, ranging from 1.6% for CSU Northridge to 21.1% for CSU Fullerton (M = 7.9%, SD = 6.6%). Finally, the distribution of marijuana-related tweets ranged from 28.6% for CSU San Marcos to 61.9% for the University of Southern California (M = 48.6%, SD = 9.60%).

**Table 1:** Proportional comparisons of product category by 4-year college, for top 20 campuses.

**College**	**Marijuana%**	**Tobacco%**	**Vaping%**	***n***	***p*** **(marijuana vs. tobacco)**	***p*** **(marijuana vs. vaping)**	***p*** **(tobacco vs. vaping)**
CSU East Bay	50.8	49.2	0.0	63	0.900	–	–
CSU Fullerton	42.1	36.8	21.1	15	0.796	0.248	0.366
CSU Humboldt	36.4	60.6	3.0	32	0.157	0.002	<0.001
CSU Long Beach	56.5	26.1	17.4	19	0.108	0.029	0.527
CSU Los Angeles	60.0	33.3	6.7	28	0.131	0.000	0.021
CSU Monterey Bay	52.2	39.1	8.7	21	0.513	0.008	0.035
CSU Northridge	41.9	56.5	1.6	61	0.249	<0.001	<0.001
CSU San Diego	52.2	30.4	17.4	40	0.105	0.005	0.201
CSU San Francisco	57.6	42.4	0.0	94	0.144	–	–
CSU San Jose	29.7	62.2	8.1	34	0.040	0.033	<0.001
CSU San Marcos	28.6	52.4	19.0	17	0.225	0.527	0.071
CSU Sonoma	47.8	39.1	13.0	20	0.655	0.033	0.083
UC Berkeley	31.8	54.5	13.6	19	0.251	0.206	0.020
UC Irvine	48.7	43.6	7.7	36	0.739	0.001	0.002
UC Los Angeles	42.9	54.3	2.9	34	0.493	<0.001	<0.001
UC Riverside	48.5	42.4	9.1	30	0.715	0.003	0.008
UC San Diego	45.8	50.0	4.2	23	0.835	0.004	0.002
UC Santa Barbara	48.9	48.9	2.1	46	1.000	<0.001	<0.001
UC Santa Cruz	52.5	42.6	4.9	58	0.431	<0.001	<0.001
USC	61.9	26.2	11.9	37	0.014	<0.001	0.134

Positive sentiment about tobacco, marijuana, and vaping was detected from 736 (67.6%) tweets. Out of the top twenty colleges by tweet volume, positive sentiment (out of all tweets with any sentiment) ranged from 55.0% for CSU Long Beach to 95.8% for CSU Los Angeles (M = 79.7%, SD = 12.0%). When computed as a proportion of all tweets with smoking-related behavior, including neutral tweets without clear user sentiment, positive sentiment ranged from 47.4% for both CSU Fullerton to 80.9% for CSU Santa Barbara (M = 71.0%, SD = 10.5%). With the exception of CSU Long Beach (47.8%) and CSU Fullerton (47.4%), all colleges had at least 50% positive sentiment from tweets about smoking (Table 2).

**Table 2:** Proportional comparisons of sentiment by 4-year college, for top 20 campuses, with *p*-value denoting test of positive vs. negative classifications.

**College**	**Positive%**	**Negative%**	**Neither%**	***n***	***p***
CSU East Bay	74.6	19.0	6.3	63	<0.001
CSU Fullerton	47.4	36.8	15.8	19	0.617
CSU Humboldt	51.5	39.4	9.1	33	0.465
CSU Long Beach	47.8	39.1	13.0	23	0.655
CSU Los Angeles	76.7	3.3	20.0	30	<0.001
CSU Monterey Bay	60.9	4.3	34.8	23	0.001
CSU Northridge	77.4	12.9	9.7	62	<0.001
CSU San Diego	64.6	25.0	10.4	48	0.004
CSU San Francisco	71.6	11.6	16.8	95	<0.001
CSU San Jose	76.3	15.8	7.9	38	<0.001
CSU San Marcos	66.7	33.3	0.0	21	0.127
CSU Sonoma	73.9	17.4	8.7	23	0.005
UC Berkeley	72.7	22.7	4.5	22	0.016
UC Irvine	55.0	27.5	17.5	40	0.056
UC Los Angeles	54.3	37.1	8.6	35	0.289
UC Riverside	78.8	15.2	6.1	33	<0.001
UC San Diego	62.5	16.7	20.8	24	0.012
UC Santa Barbara	80.9	12.8	6.4	47	<0.001
UC Santa Cruz	71.0	16.1	12.9	62	<0.001
USC	73.8	16.7	9.5	42	<0.001

Across product categories, positive sentiment varied (out of all tweets with any sentiment) with 58.2% for vaping, 66.1% for tobacco, and 70.7% for marijuana. When calculated as a proportion of all tweets, positive sentiment was 63.9% for vaping, 70.6% for tobacco, and 85.5% for marijuana. The majority of tweets from any product category exhibited either positive or negative sentiment, with only 8.9% of tweets about vaping, 6.3% about tobacco, and 17.3% about marijuana having neutral sentiment. Therefore, while the majority of tweets about any product exhibited either positive or negative sentiment, the data suggests that tweets about tobacco or vaping were much more opinionated than marijuana, which had the highest proportion of neutral sentiment tweets.

There were also 502 tweets (46.1%) denoting first-person product use or second-hand observation of another person's use of smoking products. These reports also ranged by product type, with 40.8% for marijuana, 47.4% for tobacco, and 10.0% for vaping. Out of all tweets, 40.3% of those about marijuana indicated first-person use or second-hand observation, whereas this applied to 48.6% of tobacco-related tweets and 63.3% of vaping-related tweets. Across the top twenty colleges by tweet volume, first-person smoking product use or second-hand observation of another product user ranged from 31.8% from UC Berkeley to 73.7% for CSU San Jose (M = 42.9%, SD = 12.1%).

As the UC system and CSU Fullerton had smoke-free policies in 2015, and the remaining 22 schools in the CSU system did not have smoke-free policies, these tweets were assessed for evidence relating to campus policy violation. Out of 486 tweets in 2015 indicating first-person smoking or second-hand observation of smoking, 146 (30.0%) were from schools with smoke-free policies. It should be noted that the content of these 146 tweets indicated smoking behavior on campus (e.g., “Lol the kids smoking joints and skating on campus are savage. Stupid but savage” from UC Santa Barbara). As we captured 11 schools with smoke-free policies in 2015 and 19 schools without smoke-free policies (in the public university systems), the number of these tweets per school was approximately the same among schools with smoke-free policies (13.3 per school) and those without smoke-free policies (14.2 per school), potentially indicating a muted effect regarding the implementation of smoke-free policies, at that time, on these college campus populations and their compliance behaviors.

Geospatial analysis revealed a distribution of tweets that approximately followed California's population distribution, with a cluster in the San Francisco Bay Area and a cluster in Southern California, which was dominated by the Los Angeles Basin. However, comparatively fewer smoking-related tweets were captured from colleges in California's Central Valley region. This distribution may have also been impacted by a low volume of tweets collected and sample bias for higher-population demographic areas based on the data collection process.

## Discussion

Based on our use of tweets specifically geolocated for CA 4-year universities combined with a data filtering process to isolate tweets containing smoking-related keywords, 7,342 tweets were obtained for analysis that discussed smoking and also originated from California universities between 2015 and 2019. Within this corpus of social media messages, rates for use of the term “weed” decreased over time, changing from 28% in 2015 to 7% in 2019. Other commonly used smoking-related terms did not exhibit a percentage drop of this magnitude. The mechanisms underscoring the observed decrease in social media messages with this keyword are not clear but may result from evolving word choices to describe marijuana, decreased use of marijuana on CA college campuses, social inhibition of posting marijuana-related public messages on Twitter, or some combination thereof. Further, it is unclear how passage of legalized adult-use cannabis Proposition 64 may have impacted these conversations, attitudes, and behaviors, particularly as despite state legalization, some college-aged students may not be of legal age (e.g., 21-years of age) and campus smoke free policies still restrict their use.

Manual review uncovered 1,089 tweets explicitly related to smoking behavior and posted within the boundaries of California 4-year universities, with the majority of tweets expressing positive sentiment about smoking products and behavior. Five-hundred-and-two of these tweets reported first-person use or second-hand observation of another person's smoking behavior, with 146 tweets reporting possible violations of smoke- or tobacco-free campus policies that were clearly in place from 2015 but were also in the process of being fully implemented. These tweets indicate early lack of compliance to smoke-free campus policy implementation as self-reported by social media users. For campuses where policies were not in place, tweets also reflect general positive sentiment about smoking and reports of smoking behavior, indicating possible barriers to enacting campus smoke-free policies that would occur in 2017, when more smoke free campus policies across the California State University system were enacted. These results provide early indications that smoke-free campus policy implementation requires continued attention and sufficient resources to ensure appropriate health promotion, education on policy requirements, and policy enforcement measures in college communities.

Overall, our analysis found a higher number of tweets in our corpus identified for tobacco and marijuana products, with comparatively fewer for vaping products geolocated for California university campuses. The majority of geolocated data collected during this study originated in 2015, which may explain the overemphasis on tobacco and marijuana Twitter conversations as vaping products were rising in popularity. Additionally, national debate about marijuana legalization occurred during this time frame, though was not legalized in California for adult recreational use until 2016 and licensure of cannabis retailers was permitted in 2018. As previously stated, national and state discussions relating to marijuana legalization may have influenced the relative social acceptability and volume of marijuana-related Twitter conversations among campus populations.

Tweets about vaping had the highest proportion containing first-hand accounts of use or other persons engaged in product use and behavior. The increasing popularity of vaping products throughout the study time period, especially among the college-aged population, may partly explain why college students posted about themselves or other people using vaping products in this context, despite having an overall lower volume than other smoking products (e.g., discussing options for where to vape on campus, posting pictures of vaping clouds, discussing use of new vaping products, etc.). The increasing use of more discreet forms of vaping, particularly JUUL (38), may also have had an impact on social media engagement about vaping behavior, though more research is needed. Also, the associated health risks of vaping were relatively unknown during the study period, though the outbreak of EVALI in 2019 may have generated more attention and possible concern among users about potential health risks of vaping, though these conversations were not detected in this study (39).

Importantly, most tweets that included conversations about tobacco products and behavior expressed positive sentiment. Though unclear from these preliminary results, the influence of “party culture” on college campuses, the opportunities to experiment and initiate with forms of substance abuse behavior, and the immediacy of pleasure from substance use may outweigh concerns, including those relating to long-term health risks, among college students in the United States as observed in this user sentiment (40, 41). Interestingly, though marijuana tweets exhibited the highest proportion of positive tweets, they also exhibited the highest proportion of neutral tweets and the lowest proportion of tweets with negative sentiment. This finding may suggest relative homogeneity regarding marijuana attitudes, possibly as a consequence of debate regarding marijuana legalization during this time period.

As the majority of all sentiment-containing tweets were positive, results from this study may suggest that outreach efforts to raise awareness about the health risks of tobacco and ATPs on college campuses may have limited resonance. However, these preliminary data also suggest discrepancies in sentiment between tobacco products, as well as differences in sentiment toward smoking across California universities. Therefore, policymakers and health promotion advocates should consider tailoring policy implementation and health communication for specific college students in California based upon evidence of latent receptivity toward anti-tobacco approaches and existing community sentiment toward smoking behaviors as detected in this study. Furthermore, future studies should more explicitly assess user reaction and sentiment to debate, communication and implementation of state-level policies that both legalize and restrict use of tobacco and smoking products, as well as how these macro policies interact with campus-specific smoke free policy perceptions for different tobacco, marijuana, and e-cigarette product categories.

For example, actionable insights based on preliminary findings from this study indicate that users generally express more positive sentiment about tobacco use and smoking behavior. This may necessitate the use of campus-based health promotion and education activities that focus on reducing appeal of these products, such as restricting any form of marketing and promotion in or near campus communities. This should be coupled with broader state legislation to further restrict marketing and promotion that targets young adults and college communities. Further, perceived penalties for violating smoke- and tobacco-free campus policies (with some campuses threatening academic sanctions and/or fines) may also impact compliance based on socioeconomic factors. For example, one user from UC Riverside tweeted, “other places might be more lenient, but UCs have a shitty tobacco and smoking policy and I got caught and now it's *over*” [emphasis added to denote correction of misspelling]. Hence, data-driven approaches to assess receptivity and the impact enforcement has on smoking behavior should be built into smoke free program implementation iteratively.

Importantly, the breakdown of smoking-related tweets between numerous college campuses as detected in this study presents challenges with respect to whether the distribution of tweet characteristics accurately reflects distributions in the underlying college populations. Nevertheless, similar work has been conducted which presents correlational evidence between characteristics of geospatially-specific social media posts and characteristics of populations in those areas (35, 42, 43). Furthermore, as over half of college students in California are between the ages of 18 and 24 (44), academic and demographic distributions of tobacco consumption within colleges may be the consequence of socioeconomic disparities in childhood and potential effects of these disparities on attitudes about smoking among parents, high schools, and/or neighborhoods that warrant further study (45).

Results from our study are limited in generalizability, though complement work by others on examining the impact of tobacco free policies on US college campuses. This includes a recent study from 2020 of small colleges in Massachusetts that found that a college with a smoke-free policy had significantly more anti-smoking attitude than a control campus, but did not have lower rates of smoking itself (46). Relatedly, a separate earlier study from 2005 that analyzed undergraduates in Texas found that campuses with preventive education programs had lower odds of smoking, whereas designated smoking areas and cessations programs were associated with higher odds of smoking (47). Collectively, these prior studies and our own work helps to better characterize knowledge, attitudes and behaviors of college campus communities toward smoking, as well as the smoke-free policies attempting to discourage smoking, which in turn should aid in the development of more targeted approaches to educate college-aged populations about the health harms of tobacco and also enable better implementation of anti-tobacco policies in these critical populations.

### Limitations

This study was exploratory in nature and collected social media messages for which latitude and longitude coordinates could be collected from the Twitter API, but this data collection methodology is limited to collecting messages from Twitter users that enabled geolocation, a specific limitation to generating a more generalizable dataset on Twitter as it is estimated that only 1% of all tweets are geocoded (48, 49). Hence, the dataset used in this study after filtering for keywords was small and likely biased, limiting the generalizability of results. This method of data collection may have introduced bias in the types of tweets collected, thereby limiting the generalizability of findings as the majority of Twitter users do not geolocate their posts. Potential sampling biases for Twitter include oversampling for certain geographic areas (e.g., there are a higher number of U.S. Twitter users than other countries), filtering for specific features (e.g., language, location), and the limitations of the Twitter public streaming API (used in this study) in lieu of other data collection approaches (e.g., Twitter REST and SEARCH APIs) (50). Future studies should examine the use of multiple Twitter APIs to generate a more representative Twitter dataset (including different strategies for filtering, demographic characterization, and purposeful user sampling) and compliment findings with other traditional sources of data (e.g., survey data, focus groups, clinical records, etc.) to generate findings that are more robust and generalizable, as well as use complementary Twitter and social media datasets made publicly available by other researchers. Specific to identification of Twitter users and conversations associated with colleges and universities, using keyword searches, and selecting accounts affiliated with higher education should be explored in future studies. Also, inclusion criteria required tweets to be posted from college campuses, which would not have accounted for variability in smoking-related tweets from off-campus housing or areas/neighborhoods at the borders of campus properties where college students may reside. Furthermore, though the study design permitted searches of the Twitter API to return different volume of tweets for different keywords, there was a smaller number of original keywords for substances containing marijuana/cannabis than those for e-cigarettes or products containing tobacco due to our purposeful filtering for tobacco and alternative tobacco product keywords (i.e., original keywords that captured marijuana-related tweets related to the keyword “smoking” and “weed” but we did not conduct purposeful surveillance for marijuana and cannabis specific products). Additionally, the majority of tweets analyzed for this study were from 2015, a period prior to major public scrutiny about default privacy settings for location sharing on Twitter (Twitter made a change to policy privacy settings in 2017). Finally, this study is an ecological study and should therefore be considered hypothesis-generating and not generalizable to individuals on college campuses until further studies among individuals confirm these correlational findings.

### Conclusions

Our study is exploratory and meant to generate preliminary data to inform future research and hypotheses to better elucidate tobacco and smoking knowledge, attitudes, and behaviors specific to California college communities. Overall, the sentiment of the majority of tweets detected in this study for any smoking product was positive, indicating that anti-smoking efforts on CA campuses require more targeted health promotion in order to ensure college-aged populations are fully aware of the deleterious health risks of smoking and the benefits of complying with smoke-free university policies. Study results are informative to better characterize the social media college campus climate toward smoking attitudes, behaviors, and non-compliance with university smoking policies. More research is needed to better understand how college populations respond to different campus anti-smoking initiatives, ideally through the use of mixed research approaches including quantitative, qualitative, and also infoveillance methods as explored in this study.

## Data Availability Statement

The data that support the findings of this study are available from the corresponding author, Raphael E. Cuomo, upon reasonable request.

## Author Contributions

NO and JL collected social media data for this study. VP, CB, MN, and NS conducted manual annotation of social media data. RC conducted geospatial and statistical analysis. All authors contributed to the conceptualization and design of the study, drafted the manuscript, and approved the final version.

## Conflict of Interest

JL, CB, MN, and TM are employees of the startup company S-3 Research LLC. S-3 Research is a startup funded and currently supported by the National Institutes of Health—National Institute on Drug Abuse through a Small Business Innovation and Research contract for opioid-related social media research and technology commercialization. The remaining authors declare that the research was conducted in the absence of any commercial or financial relationships that could be construed as a potential conflict of interest.

## Appendix

**Table A1:** Manual annotation scheme used for trinary classification of sentiment and binary classification of first-person use or second-hand observation of smoking behavior.

**Signal**	**Sentiment**	**Coding scheme**
Yes	Positive	• Tweets reporting first-person/self-use of smoking/vaping products/marijuana <or> • Tweets reporting cigarette smoking/vaping/marijuana smoking by others (second-hand observation) <and> • Expressing favorable sentiment about smoking/vaping and being encouraging of the behavior • Expressing compliments toward smoking behavior or advocate use of tobacco products • Expressing potential future use of tobacco and/or marijuana products • Advocating smoking as a personal choice and expressing disagreement with campus smoking ban policies
	Negative	• Tweets reporting second-hand observation of smoking or vaping <or> • Tweets reporting second-hand marijuana smoking <and> • Expressing disgust/discomfort of being in the vicinity of those smoking/vaping • Expressing concern about second-hand smoke inhalation or passive smoking • Expressing disappointment/anger about campus smoking bans being defied
	Neutral	• Tweets reporting second-hand observation of smoking without expressing opinions for or against the behavior
No	–	• Tweeting news posts related to smoking products/smoking behavior • Discussing adverse health effects of smoking to create awareness • Discussing smoking behaviors of celebrities/other public figures • Retweeting policies related to campus smoking bans • Sarcasm/jokes on smoking products or smoking behavior • Tweets unrelated to smoking products or smoking behavior (“noise”)

*Manual review also collected a trinary classification of product type used (i.e., marijuana, tobacco, and/or vaping) for posts specifying a product type*.
